# BOLD signal and functional connectivity associated with loving kindness meditation

**DOI:** 10.1002/brb3.219

**Published:** 2014-02-12

**Authors:** Kathleen A Garrison, Dustin Scheinost, R Todd Constable, Judson A Brewer

**Affiliations:** 1 Department of Psychiatry, Yale University School of MedicineNew York, New York; 2 Department of Diagnostic Radiology, Yale University School of MedicineNew Haven, Connecticut

**Keywords:** Connectivity, default mode network, fMRI, loving kindness, meditation, metta

## Abstract

Loving kindness is a form of meditation involving directed well-wishing, typically supported by the silent repetition of phrases such as “may all beings be happy,” to foster a feeling of selfless love. Here we used functional magnetic resonance imaging to assess the neural substrate of loving kindness meditation in experienced meditators and novices. We first assessed group differences in blood oxygen level-dependent (BOLD) signal during loving kindness meditation. We next used a relatively novel approach, the intrinsic connectivity distribution of functional connectivity, to identify regions that differ in intrinsic connectivity between groups, and then used a data-driven approach to seed-based connectivity analysis to identify which connections differ between groups. Our findings suggest group differences in brain regions involved in self-related processing and mind wandering, emotional processing, inner speech, and memory. Meditators showed overall reduced BOLD signal and intrinsic connectivity during loving kindness as compared to novices, more specifically in the posterior cingulate cortex/precuneus (PCC/PCu), a finding that is consistent with our prior work and other recent neuroimaging studies of meditation. Furthermore, meditators showed greater functional connectivity during loving kindness between the PCC/PCu and the left inferior frontal gyrus, whereas novices showed greater functional connectivity during loving kindness between the PCC/PCu and other cortical midline regions of the default mode network, the bilateral posterior insula lobe, and the bilateral parahippocampus/hippocampus. These novel findings suggest that loving kindness meditation involves a present-centered, selfless focus for meditators as compared to novices.

## Introduction

Loving kindness (*metta*) meditation is a contemplative practice considered to promote a state of acceptance and compassion for the self and others (Gunaratana 2002). Loving kindness is practiced by directed well wishing, typically supported by silent repetition of phrases such as “may X be happy.” In so doing, practitioners cultivate openness, present-centered awareness, and selfless love, toward themselves and others (Salzberg 1995). Loving kindness and related practices such as compassion meditation have been found to enhance positive and diminish negative emotional states, and have shown preliminary utility in the treatment of depression, social anxiety, and stress, among others (for review see Hofmann et al. 2011). Yet little is known about the neural substrate of loving kindness meditation.

Related studies have assessed the effects of loving kindness or compassion meditation on the neural response to cognitive or affective tasks. For example, a recent study (Lee et al. 2012) reported that loving kindness meditation led to changes in the neural response to viewing emotional faces, in brain regions implicated in emotion processing, including the left ventral anterior cingulate cortex, right inferior frontal gyrus (IFG), and right precuneus for happy faces, and the left caudate and middle frontal gyrus for sad faces. Another study (Lutz et al. 2008) found that compassion meditation led to increased activation in brain regions involved in affective processing in response to emotional sounds, including the dorsal anterior cingulate cortex (dACC) and insula. Another recent study (Weng et al. 2013) found that compassion meditation training led to increased altruistic behavior outside of the training context, and associated changes in the neural response to suffering during post-pre functional magnetic resonance imaging (fMRI) in brain regions involved in social cognition and emotion regulation, including the inferior parietal cortex and dorsolateral prefrontal cortex. These neuroimaging studies provide evidence that loving kindness and related meditation practices can alter emotional or affective processing, yet do not describe the neural underpinnings of loving kindness meditation without a concurrent task. Thus, the aim of this study was to assess the neural substrate of loving kindness meditation.

A prior study from our research group, which was designed to test for common neural activation patterns across three meditation types (Brewer et al. 2011), found that loving kindness led to reduced blood oxygen level-dependent (BOLD) signal in clusters in the inferior temporal gyrus/uncus/amygdala, posterior cingulate cortex/precuneus (PCC/PCu), and the inferior parietal lobule, in experienced meditators as compared to novices. Moreover, relatively reduced BOLD signal in meditators in the PCC/PCu—a hub of the default mode network (DMN) involved in self-related processing and mind wandering (Northoff et al. 2006)—was common across all three meditation types. This study investigates the neural substrate of loving kindness meditation in a larger sample size of meditators and novices. In particular, we were interested in whether loving kindness meditation is associated with reduced BOLD signal and functional connectivity in brain regions involved in self-referential processing, in meditators as compared to novices. We first assessed group differences in BOLD signal during loving kindness meditation. We next used a relatively novel approach, the intrinsic connectivity distribution (ICD) of functional connectivity, to identify regions that differ in intrinsic connectivity between groups. On the basis of our prior interest in the PCC/PCu, we then used a data-driven approach to seed-based connectivity analysis to identify which connections with this brain region differ between groups during loving kindness.

## Material and Methods

### Participants

Twenty experienced meditators (11 men, 9 women, 20 white non-Hispanic, mean age 45.6 years, mean education 17.6 years) and 26 novices (15 men, 11 women, 26 white non-Hispanic, mean age 42.2 years, mean education 17.2 years) took part in the study. Groups were matched by gender (*χ*^2^ = 0.03, *P* = 0.85), age (*t* = −0.92, *P* = 0.36), and education (*t* = −0.36, *P* = 0.72). Meditators were drawn from the Theravada/insight meditation tradition, and reported a total of 9675 ± 1586 (mean ± standard error of the mean; standard deviation = 7092) practice hours over 14 ± 2 years, consisting of both daily practice and retreats. Meditators also reported a total of 752 ± 217 practice hours of loving kindness meditation over 9 ± 2 years. All meditators were experienced in loving kindness meditation. Novices reported no prior meditation experience. All participants provided written informed consent in accordance with the Declaration of Helsinki and the institutional review board of Yale University.

### Meditation instructions

Participants were instructed in three standard mindfulness meditation practices: loving kindness, concentration, and choiceless awareness (Gunaratana 2002; Brewer et al. 2011). This analysis is focused only on the loving kindness meditation condition. Loving kindness instructions were: “Please think of a time when you genuinely wished someone well (pause). Using this feeling as a focus, silently wish all beings well, by repeating a few short phrases of your choosing over and over. For example: May all beings be happy, may all beings be healthy, may all beings be safe from harm.” Participants were instructed to meditate with their eyes closed. Participants practiced loving kindness meditation outside of the scanner and confirmed that they understood and could follow the instructions.

### fMRI task

Each run began with a 30-sec eyes open baseline. This state was followed by an 8-sec slide reminding participants of the active baseline instructions and a 90-sec active baseline, during which participants viewed words and decided whether or not the words described them, or whether or not the words were in upper case letters, or rested (Kelley et al. 2002). The active baseline task was followed by a 30-sec eyes closed baseline. This state was followed by a 30-sec recorded meditation instruction (as above) and a 180-sec meditation period. Each run ended with an additional approximately 20-sec eyes open baseline. Each meditation condition was performed twice. Meditation conditions were presented in random order, but the second instance of each was blocked (i.e., AABBCC). After each run, participants were asked to rate how well they were able to follow the instructions and how much their mind wandered on a scale from 0 to 10.

### Imaging data acquisition

Images were obtained with a Siemens 1.5 Tesla Sonata MRI system (Siemens AG, Erlangen, Germany) using a standard eight-channel head coil. High-resolution T1-weighted 3D anatomical images were acquired using a magnetization prepared rapid gradient echo sequence (time to repetition [TR] = 2530 msec, time to echo [TE] = 3.34 msec, field of view = 220 mm, matrix size = 192 × 192, slice thickness = 1.2 mm, flip angle = 8°, with 160 slices). Low-resolution T1-weighted anatomical images were then acquired (TR = 500 TE = 11 msec, field of view = 220 mm, slice thickness = 4 mm, gap = 1 mm, 25 AC-PC aligned axial-oblique slices). Functional image acquisition began at the same slice location as the T1 scan. Functional images were acquired using a T2*-weighted gradient-recalled single-shot echo-planar sequence (TR = 2000 msec, TE = 35 msec, flip angle = 90°, bandwidth = 1446 Hz/pixel, matrix size = 64 × 64, field of view = 220 mm, voxel size = 3.5 mm, interleaved, 210 volumes, after 2 volumes were acquired and automatically discarded).

### Imaging data preprocessing

Images were preprocessed using SPM8 (http://www.fil.ion.ucl.ac.uk/spm). Functional images were realigned for motion correction and resultant parameters were used as regressors of no interest in the fMRI model. Artifact Detection Tools (ART; http://www.nitrc.org/projects/artifact_detect) was used to identify global mean intensity and motion outliers in the fMRI time series, and any detected outliers were included as regressors of no interest in the fMRI model. The structural image was coregistered to the mean functional image and segmented. All images were normalized to the Montreal Neurological Institute (MNI) template brain using SPM8 unified segmentation normalization (Ashburner and Friston 2005), and smoothed using a 6 mm full width at half-maximum Gaussian kernel.

### General linear model analysis

Blood oxygen level-dependent signal was modeled using separate regressors for the conditions: eyes open baseline, active baseline instruction, active baseline, meditation instruction, and meditation. Eyes closed state was included as implicit baseline. Conditions were modeled using a boxcar function convolved with a canonical hemodynamic response function, and fit using SPM8's implementation of the general linear model (GLM). For this analysis, first level maps were generated for loving kindness meditation relative to implicit baseline. Second level maps were generated to compare between groups, meditators and novices, using independent *t*-tests. Second level maps were thresholded at *P* < 0.05 family wise error (FWE), cluster corrected for multiple comparisons, with a cluster-forming threshold of *P* < 0.05. For all reports, functional activation was localized based on cytoarchitectonic probability maps using SPM Anatomy toolbox (Eickhoff et al. 2005), and Brodmann areas were assigned based on ≥30% probability.

### Functional connectivity analysis

For functional connectivity analysis, only the long (180-sec) loving kindness meditation blocks were used for analysis, similar to prior task-based connectivity analyses of meditation blocks (Brewer et al. 2011). First, additional preprocessing was performed using CONN toolbox (Whitfield-Gabrieli and Nieto-Castanon 2012) to model realignment parameters and BOLD signal from the white matter and cerebrospinal fluid masks as covariates of no interest, using CompCor component-based noise correction (Behzadi et al. 2007).

After preprocessing, the two loving kindness runs were concatenated, and voxel-to-voxel connectivity was measured using the ICD method in Bioimage suite (Joshi et al. 2011; http://www.bioimagesuite.org). ICD measures a voxel's “average” connectivity to every other voxel in the brain. Like other voxel-to-voxel connectivity measures (e.g., Buckner et al. 2009), ICD treats each voxel in the gray matter as a seed and computes standard seed connectivity at this voxel. The resulting seed connectivity map is then summarized into a single number using graph theory. First, this map is converted to a histogram of correlations used to estimate the distribution of connections to the seed voxel. Second, this distribution is modeled as a Weibull distribution with the variance used as the parameter of interest. The Weibull distribution is fitted to the data using standard regression analysis. A larger variance indicates a greater number of high correlation connections, and thus greater connectivity. The variance of this distribution has been shown to be more sensitive to group differences than simpler parameters such as the mean. This method was repeated for all gray matter voxels to derive a parametric map for each participant where each voxel represents a voxel's correlation to the rest of the brain. Voxel-wise, two-sample *t*-tests were used to compare ICD during loving kindness meditation between groups, meditators, and novices. Because ICD was measured for the loving kindness condition only, with a block length of 180 sec, we did not weight the model related to any potential effects of task-switching. Second level maps were thresholded at *P* < 0.05 FWE, cluster corrected using a cluster-forming threshold of *P* < 0.005.

Seed-to-voxel connectivity was then measured using CONN toolbox. A seed region was defined in our a priori region of interest, the PCC/PCu, as a 10 mm sphere centered on the peak voxel in that brain region in the between-subject ICD map (MNI coordinates: 18, −54, 18; Table S1). The seed mask was generated using MarsBar (http://marsbar.sourceforge.net). The BOLD timecourse was extracted from the seed region, and Pearson's correlation coefficients were computed between that timecourse and every other voxel in the brain. Correlation coefficients were converted using Fisher's transform to normally distribute scores for second level analysis, to compare between groups, meditators and novices. Second level maps were thresholded at *P* < 0.05 FWE, cluster corrected using a cluster-forming threshold of *P* < 0.05.

Finally, although motion outliers were removed using ART, group differences in motion may influence functional connectivity results. We thus calculated average frame-to-frame displacement for each group (Van Dijk et al. 2012). We found no significant difference in motion between meditators and novices (*T* = 0.23; *P* = 0.8192).

### Statistical analysis

Statistical analyses were conducted using SPSS 19.0 (http://www-01.ibm.com/software/analytics/spss/software). For demographics, independent sample *t*-tests were used to determine differences between meditators and novices in age and years of education, and chi-square tests were used to determine differences between meditators and novices in gender. For self-report, multivariate analysis of variance was used to determine differences between meditators and novices in mind wandering. All tests of significance are reported as two-sided as means ± standard deviation.

## Results

### Self-report

Meditators reported less mind wandering during meditation relative to novices across all meditation conditions (*F*_(1,44)_ = 7.57, *P* = 0.009), and this finding was apparent for loving kindness meditation (novices = 3.8 ± 1.8, meditators = 2.8 ± 1.4). In addition, both meditators and novices reported being able to follow the instructions to a high degree for loving kindness meditation (novices = 8.6 ± 1.4, meditators = 8.8 ± 1.2).

### Main effect of loving kindness meditation

**Figure 1:**
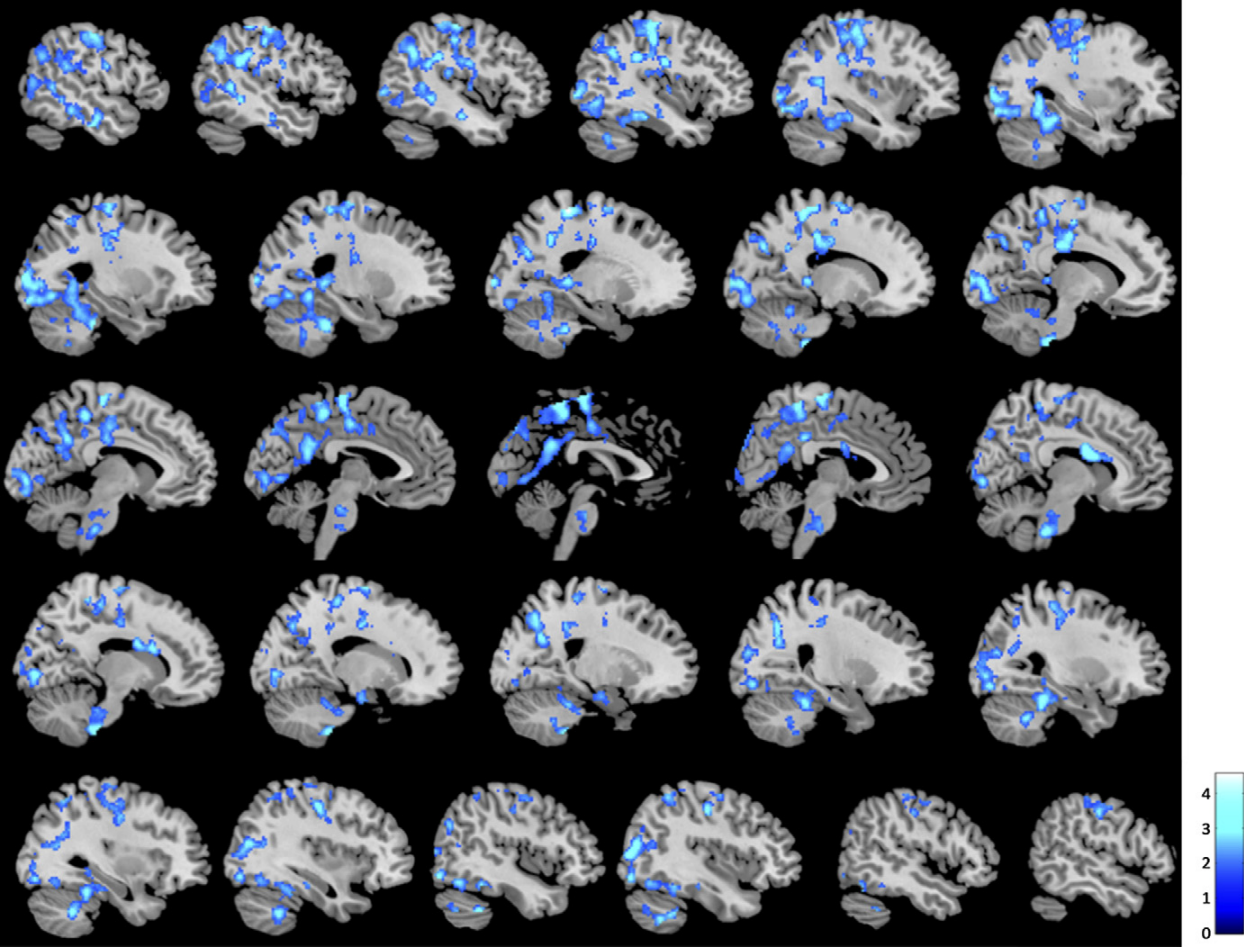
Brain regions showing reduced BOLD signal during loving kindness meditation in meditators as compared to novices (*P* < 0.05 FWE, cluster corrected; slices displayed left to right).

Whole brain contrast maps revealed a significant difference in BOLD signal during loving kindness meditation between meditators and novices, such that meditators showed less BOLD signal in clusters including the left postcentral gyrus, inferior parietal lobule and precentral gyrus; left supramarginal gyrus; left angular gyrus; left middle and superior temporal gyrus; left middle cingulate cortex (MCC); left posterior cingulate cortex and bilateral precuneus; bilateral postcentral gyrus, right supplementary motor area, left paracentral gyrus and left MCC; bilateral temporal gyrus, fusiform gyrus, occipital gyrus and cerebellum; left hippocampus; and right precentral gyrus (Fig. 1; all slices displayed in Fig. S1). No regions showed greater BOLD signal in meditators compared to novices.

### Intrinsic connectivity distribution

**Figure 2:**
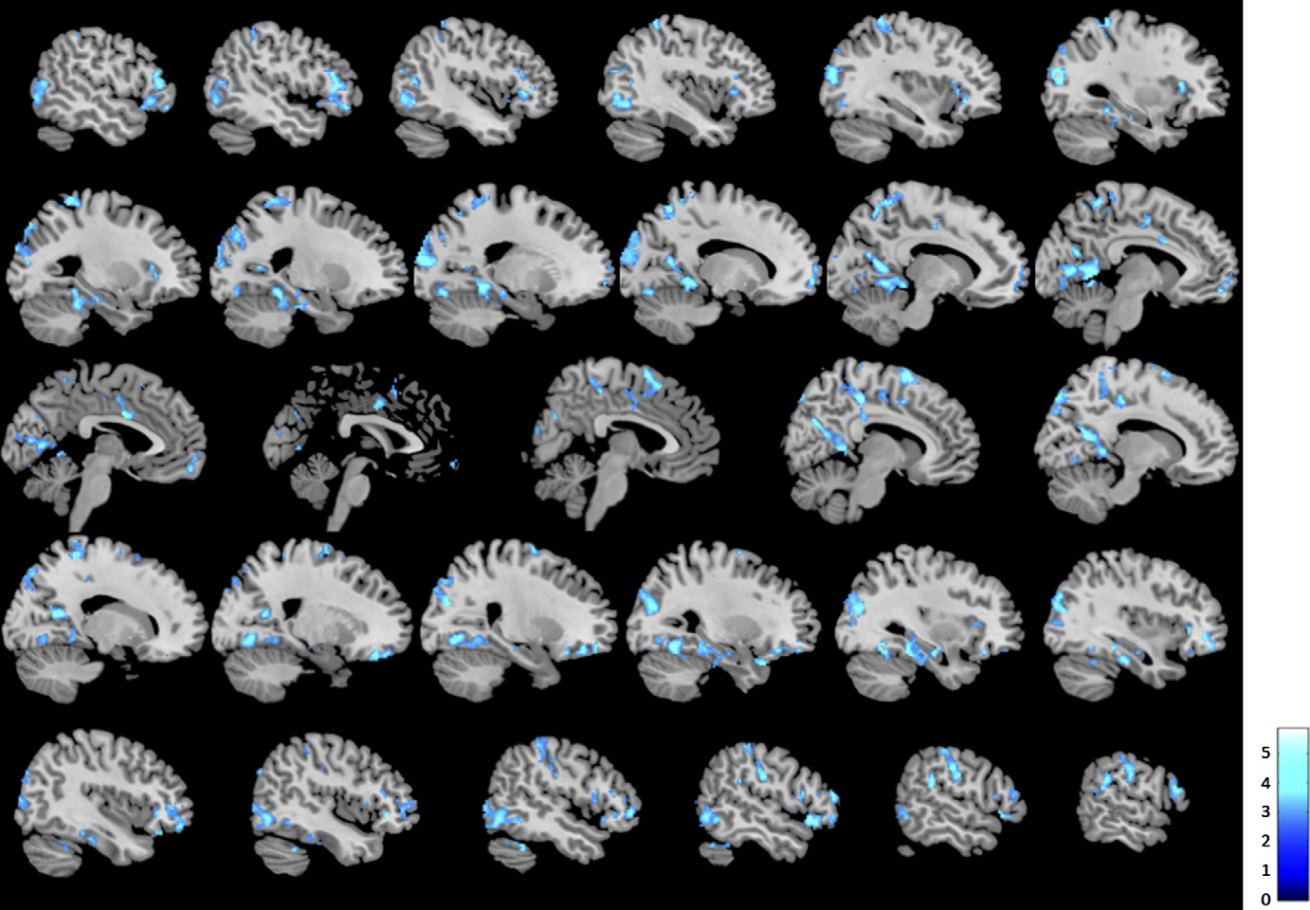
Brain regions showing less intrinsic connectivity during loving kindness meditation in meditators as compared to novices (*P* < 0.05 FWE, cluster corrected; slices displayed left to right).

Whole-brain contrast maps showed a significant difference in ICD during loving kindness meditation between meditators and novices, such that meditators showed less ICD as compared to novices overall, and in clusters including the right fusiform gyrus and precuneus; right supramarginal gyrus and temporal gyrus; bilateral occipital gyrus, temporal gyrus, and parietal lobule; bilateral precuneus, postcentral gyrus, and parietal lobule; MCC and dACC; bilateral supplementary motor area; bilateral IFG and insula lobe; bilateral parahippocampal gyrus; and left superior orbital gyrus (Fig. 2, Table S1; all slices displayed in Fig. S2). No regions showed greater ICD in meditators compared to novices.

### Seed-based functional connectivity

**Figure 3:**
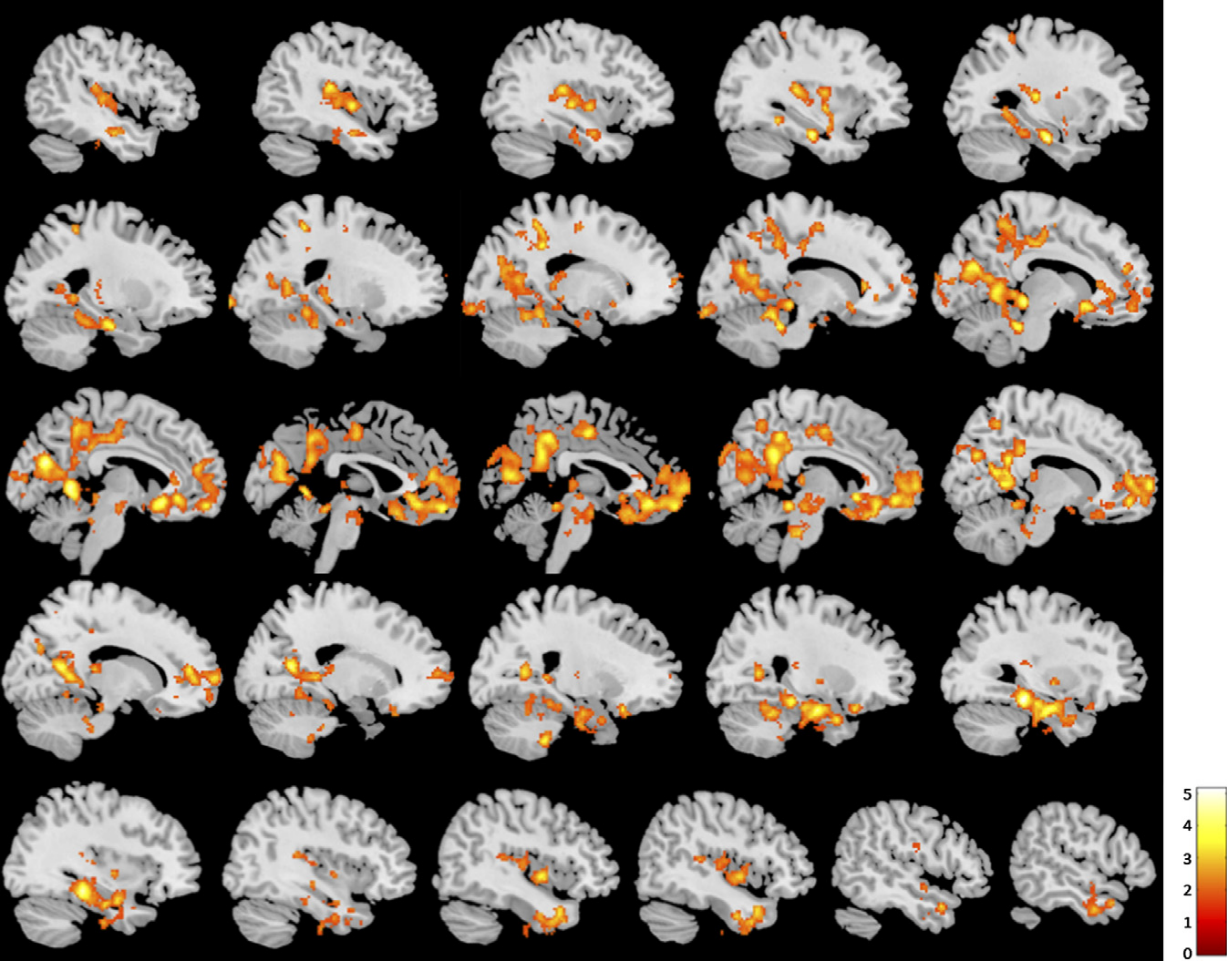
Brain regions showing greater functional connectivity with the posterior cingulate cortex/precuneus during loving kindness meditation in novices than meditators (*P* < 0.05 FWE, cluster corrected; slices displayed left to right).

**Figure 4:**
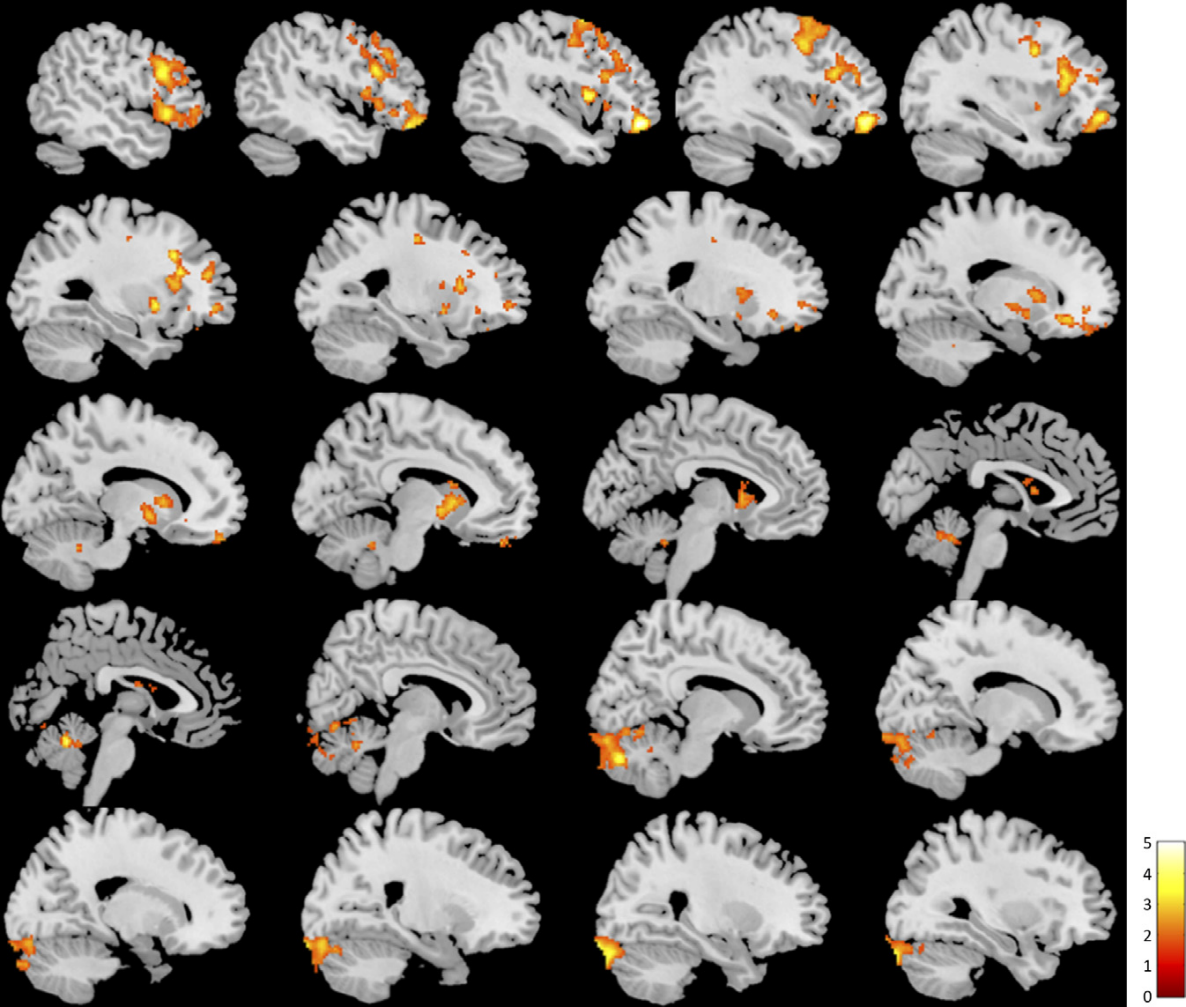
Brain regions showing greater functional connectivity with the posterior cingulate cortex/precuneus during loving kindness meditation in meditators than novices (*P* < 0.05 FWE, cluster corrected; slices displayed left to right).

Whole-brain contrast maps revealed a significant difference in functional connectivity with the PCC/PCu during loving kindness meditation between meditators and novices. Novices showed greater functional connectivity between the PCC/PCu and clusters in the bilateral parahippocampal gyrus, hippocampus, cerebellum, precuneus, posterior cingulate cortex, and posterior insula lobe; and the bilateral middle orbital gyrus, anterior cingulate cortex, and superior medial gyrus (Fig. 3, Table S2; all slices displayed in Fig. S3). Meditators showed greater functional connectivity between the PCC/PCu and clusters in the left IFG, middle frontal gyrus and insula lobe, and the right cerebellum (Fig. 4, Table S3; all slices displayed in Fig. S4).

## Discussion

This fMRI study describes the neural substrate of loving kindness meditation in meditators as compared to novices. To our knowledge, no prior neuroimaging study has reported on the neural substrate of loving kindness without a concurrent task. In addition to GLM analyses, we used a relatively novel method, the ICD, to identify regions of the brain that differ in the degree of connectivity between groups during loving kindness meditation. On the basis of our prior interest in the PCC/PCu, we used secondary seed-based analysis to identify which connections with this brain region differed between groups during loving kindness meditation. Overall, meditators showed reduced BOLD signal and intrinsic connectivity during loving kindness meditation as compared to novices.

From a practical perspective, loving kindness meditation involves an offering of well-wishing toward oneself and others, often described as a heartfelt feeling of selfless love (*agape*) (Salzberg 1995; Gunaratana 2002). Teachings in loving kindness rely on examples to convey this quality, such as “imagine meeting a dear friend who you haven't seen in a long time, and pay attention to the heartfelt feeling that arises in your chest.” Novices are taught to attend to this feeling, and to foster the feeling by repeating phrases of well-wishing (“may you be happy”). This is considered to help novices to remain on task and to allow the feeling of loving kindness to arise and stabilize. As practice develops and novices are able to bring about the feeling of loving kindness, the phrases may be dropped to allow one's attention to rest in the feeling itself. Loving kindness is considered a non-self-referential practice; rather than one's “self” offering well-wishes to “others,” loving kindness is offered from a condition of selflessness, for the benefit of all (Salzberg 1995).

In this study, the main effect of loving kindness differed between meditators and novices, such that meditators showed less BOLD signal than novices during loving kindness meditation in clusters including the PCC/PCu; the left MCC; and the left supramarginal gyrus, angular gyrus, middle and superior temporal gyrus; among others. With regard to group differences in BOLD signal in the PCC/PCu, these findings are consistent with our prior work (Brewer et al. 2011) suggesting this region may be a hub of the DMN that is relatively less active in meditators as compared to novices across meditation practices, including loving kindness. The PCC/PCu has been implicated as a region of the DMN involved in self-referential processing and mind wandering (Northoff et al. 2006; Buckner et al. 2008). Less activity in this brain region during meditation may reflect less self-related thinking and mind wandering (among others; see Garrison et al. 2013). These findings support the theoretical perspective that loving kindness is a focused and/or present-centered practice similar to other forms of meditation such as breath awareness (Gunaratana 2002), and that loving kindness involves a non-self focus (Salzberg 1995). One possible interpretation of the group difference in the PCC/PCu is that novices may practice directed well-wishing in loving kindness from a stance of duality, that is, “self” directing well-wishes toward “other,” whereas meditators have learned to practice “selfless” well-wishing. With regard to group differences in BOLD signal in the left parietal and temporal cluster, the left temporal parietal junction (TPJ) is considered a node of the DMN (Andrews-Hanna et al. 2010), and has been implicated in theory of mind (e.g., Samson et al. 2004). Recent studies have suggested that the left TPJ is particularly involved in processing socially relevant information about others (Saxe and Wexler 2005; Ciaramidaro et al. 2007). Reduced activations in the TPJ during loving kindness in meditators as compared to novices may reflect less self-related thinking and mentalizing (Frith and Frith 2006). Prospective studies following novices over the course of training in loving kindness are needed to test these interpretations.

Further group differences were found in the ICD of functional connectivity between meditators and novices during loving kindness meditation. A difference in ICD indicates that a given brain region shows altered connectivity to the rest of the brain on average. Here, meditators showed less ICD than novices overall, and in clusters including the bilateral IFG and insula; MCC and dACC; the PCu; and the right supramarginal gyrus and temporal gyrus.

With regard to group differences in ICD in the right parietal and temporal regions, the right TPJ has been implicated in theory of mind and empathy, including the attribution of mental states (Saxe and Wexler 2005), the sense of agency, and reorienting attention to salient stimuli (Decety and Lamm 2007). Less ICD in this brain region during loving kindness in meditators as compared to novices may again reflect less self-related processing or mentalizing, or possibly a difference in attentional processes between groups. However, such interpretations would need to be tested by comparing loving kindness with for example a mentalizing task.

With regard to group differences in intrinsic connectivity in the bilateral IFG and insula, the IFG has been implicated in emotion processing, from emotional feeling to emotion simulation and empathy (Jabbi and Keysers 2008; Shamay-Tsoory et al. 2009). Prior studies of the effects of loving kindness or compassion meditation on emotion processing have reported changes in the IFG and anterior insula (Lutz et al. 2008; Lee et al. 2012; Weng et al. 2013). For example, a recent study found that compassion meditation training led to improved empathic accuracy on the ‘Reading the Mind in the Eyes Test’ in which subjects are asked to infer others' mental states from viewing their eyes, and this was associated with greater BOLD signal in the bilateral IFG (Mascaro et al. 2013). Related to this, group differences in ICD were found in the MCC and dACC. A recent meta-analysis of fMRI studies of empathy found consistent activations in the anterior middle cingulate cortex (aMCC), dACC, supplementary motor area, and anterior insula/IFG, with the aMCC more frequently reported in studies of cognitive-evaluative empathy, where subjects are explicitly instructed to evaluate others' emotional or sensory states (Fan et al. 2011). Related to the current findings, it is possible that novices engage in more emotional processing related to empathy during loving kindness meditation than meditators.

Another interpretation is that meditators rely less on language processing during loving kindness, given that the left IFG is considered the neuroanatomical basis of inner speech (e.g., McGuire et al. 1996), including inner speech related to self-referential processing (Morin and Michaud 2007). For example, a recent fMRI study showed that a form of mantra meditation led to greater BOLD signal in the bilateral IFG than a concentration meditation task (Davanger et al. 2010). It is possible that novices practice loving kindness with a greater reliance on inner speech (“may X be happy”), whereas meditators rest more in an embodied feeling of loving kindness. As noted above, loving kindness practice initially relies on the silent repetition of phrases to generate the feeling of loving kindness, and as practice develops, the phrases may be dropped to rest in the feeling itself. This may be reflected in the group differences found in the IFG in this study, but should be tested across loving kindness training, and would be bolstered by self-report of this change in cognitive strategy.

On the basis of the current findings and our previous work, we then measured seed-based connectivity with the PCC/PCu, to investigate functional connectivity with this brain region implicated in self-related processing. Although our prior study (Brewer et al. 2011) used a PCC seed derived from the literature, this study used a data-driven approach, by seeding a sphere around the peak voxel in the PCC/PCu that differed in ICD between meditators and novices during loving kindness meditation. In this way, we first identified the group difference in ICD in this a priori region of interest during loving kindness, and we then determined which specific connections with this brain region differed between groups. We found that meditators showed greater functional connectivity during loving kindness than novices between the PCC/PCu and the left IFG. One interpretation of this finding is that when the mind wanders, meditators return to reliance on the silent repetition of phrases, or to emotional processing or empathy, to reground themselves in the feeling of loving kindness, hence increased coincident activity between the PCC/PCu and the left IFG. This interpretation would be bolstered by a neuroimaging study with self-report in which meditators' report that they indeed use the phrases to reground their practice in this way.

In contrast, novices showed greater functional connectivity than meditators between the PCC/PCu and other cortical midline structures including the medial prefrontal cortex (MPFC), anterior cingulate cortex (ACC); and the bilateral parahippocampus/hippocampus. The PCC/PCu and MPFC are hubs of the DMN with functional connections to all other DMN regions (Buckner et al. 2008), and taken together with the ACC are the regions most consistently implicated in self-related processing (Northoff et al. 2006; Qin and Northoff 2011). The parahippocampal cortex and hippocampal formation are also considered components of the DMN (Andrews-Hanna et al. 2010). Many studies have shown meditation effects in the hippocampus, most studies reporting structural changes such as increased gray matter volume (Holzel et al. 2008; Luders et al. 2009, 2013), including in the right parahippocampal gyrus of meditators who are experts at loving kindness (Leung et al. 2013). Functional neuroimaging studies have also implicated the parahippocampus/hippocampus in meditation (e.g., Lazar et al. 2000), including a form of mantra meditation (Engstrom et al. 2010). It is thought that repeated activation of the parahippocampus/hippocampus during meditation may lead to structural changes (Holzel et al. 2008). In those studies, meditation was considered to alter activity in the hippocampus related to the modulation of cortical arousal and responsiveness (Newberg and Iversen 2003; Holzel et al. 2008). Another possible interpretation of the current findings is that novices rely more on memory and emotional memory processes during loving kindness than meditators, and come back to memory processes upon mind wandering, hence greater coincident activation between the PCC/PCu and the parahippocampus/hippocampus. The instructions for loving kindness meditation in traditional practice (and in this study) ask one to: “Think of a time when you genuinely wished someone well.” In the same way that meditators, with practice, rely less on the repetition of phrases to generate the feeling of loving kindness, they may, as practice develops, rely less on memory processes to generate loving kindness. Again, prospective studies measuring changes in the neural substrate across loving kindness training are needed to test these interpretations.

This study describes the neural substrate of loving kindness meditation in a large sample of meditators and novices. Multiple neuroimaging analysis methods were used to identify differences in BOLD signal and functional connectivity between groups. Our findings indicate that novices and meditators engage different brain regions during loving kindness meditation, and provide insight into differences in cognitive strategy between groups. Novices more strongly engage brain regions involved in empathy and social cognition, inner speech, and memory processes, as well as more generally regions involved in self-related processing or mind wandering. Meditators engage these brain regions less than novices, consistent with the perspective that loving kindness meditation involves a present-centered and selfless focus.

Several aspects of this study design limit these interpretations. By comparing meditators to novices, it is possible that group differences in this study reflect preexisting differences in individuals drawn to meditation practice. It is also possible that group differences reflect state-dependent changes from long-term meditation experience, including changes that are not specific to loving kindness practice. Here, meditators reported experience with loving kindness as number of hours of practice. This is a relatively crude assessment, though a current standard in the field due to the lack of objective measures of proficiency (for review see Awasthi 2012). In particular, retrospective recall as a means for calculating the number of hours of loving kindness practice over years is problematic. Because loving kindness overlaps with other forms of meditation from the Theravada/insight tradition in the practice of “letting go” of the conceptual self, it is likely and difficult to ascertain how experience in these other practices influences loving kindness. Even with a smaller number of reported hours of loving kindness, meditators may practice loving kindness in a selfless manner as compared to novices based on their many hours spent practicing other forms of meditation from this tradition. This finding may be of particular relevance to understanding the commonalities between different meditation practices, and is supported empirically by the findings of this study. These confounds can be addressed in studies comparing the neural substrate of different meditation practices in meditators; this study was underpowered to make such comparisons. Future studies should also track changes in the neural substrate of loving kindness and other meditation practices across training. Future work may also test the relationship between these findings and behavioral or clinical measures such as compassionate behavior (Condon et al. 2013).

## Supporting Information

**Figure S1.** All slices are displayed for the comparison of BOLD signal during loving kindness meditation between meditators and novices, corresponding to Fig. 1. Brain regions in blue/cold show reduced BOLD signal during loving kindness meditation in meditators as compared to novices (*P* < 0.05 FWE, cluster corrected; slices displayed left to right).

**Figure S2.** All slices are displayed for the comparison of intrinsic connectivity during loving kindness meditation between meditators and novices, corresponding to Fig. 2. Brain regions in blue/cold show less intrinsic connectivity during loving kindness meditation in meditators as compared to novices (*P* < 0.05 FWE, cluster corrected; slices displayed left to right).

**Figure S3.** All slices are displayed for the comparison of seed-based functional connectivity during loving kindness meditation for novices greater than meditators. Brain regions in yellow/hot show greater functional connectivity with the posterior cingulate cortex/precuneus during loving kindness meditation in novices than meditators (*P* < 0.05 FWE, cluster corrected; slices displayed left to right).

**Figure S4.** All slices are displayed for the comparison of seed-based functional connectivity during loving kindness meditation for meditators greater than novices. Brain regions in yellow/hot show greater functional connectivity with the posterior cingulate cortex/precuneus during loving kindness meditation in meditators than novices (*Ps* < 0.05 FWE, cluster corrected; slices displayed left to right).

**Table S1.** Brain regions showing less intrinsic connectivity during loving kindness meditation in meditators as compared to controls.

**Table S2.** Brain regions showing greater functional connectivity with the posterior cingulate cortex/precuneus during loving kindness meditation in novices as compared to meditators.

**Table S3.** Brain regions showing greater functional connectivity with the posterior cingulate cortex/precuneus during loving kindness meditation in meditators as compared to novices.
