# Supplementary figures and images for: BOLD signal and functional connectivity associated with loving kindness meditation
Source: Brain Behav. 2014 Feb 12;4(3):337–47. doi: 10.1002/brb3.219 (PMC4055184; doi:10.1002/brb3.219)

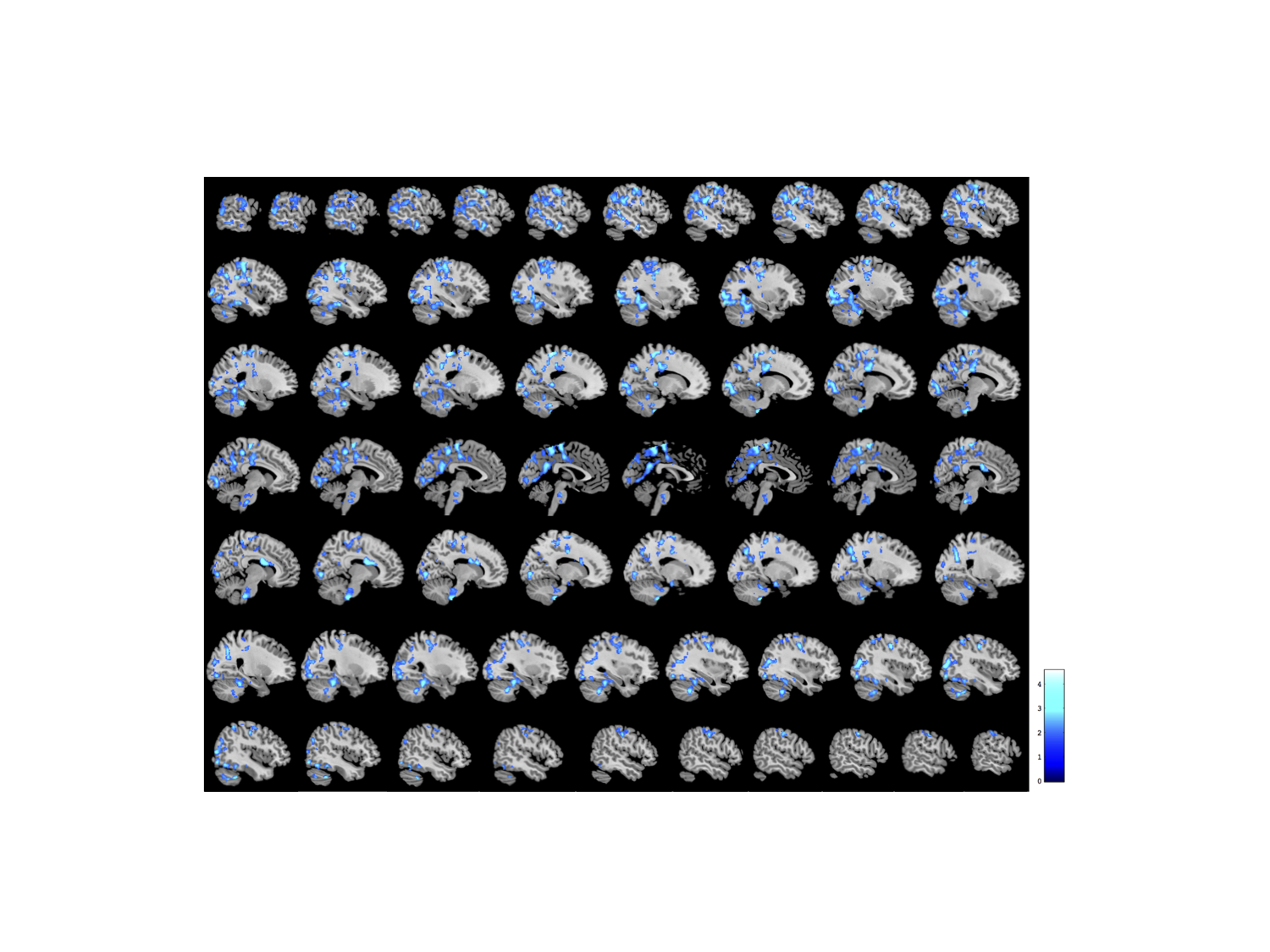

Supplement: Supplementary file 1 [file brb30004-0337-SD1.tiff]

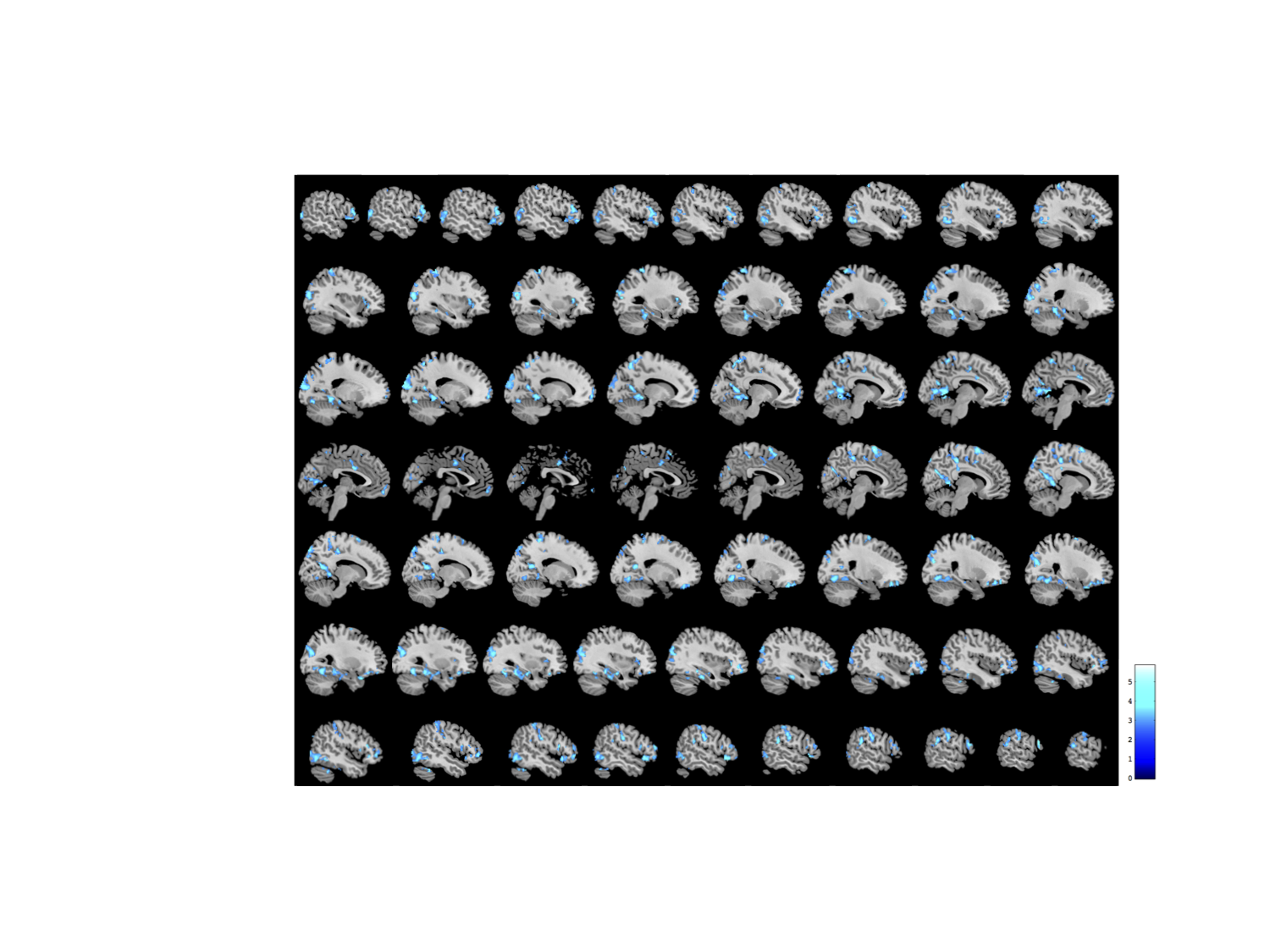

Supplement: Supplementary file 2 [file brb30004-0337-SD2.tiff]

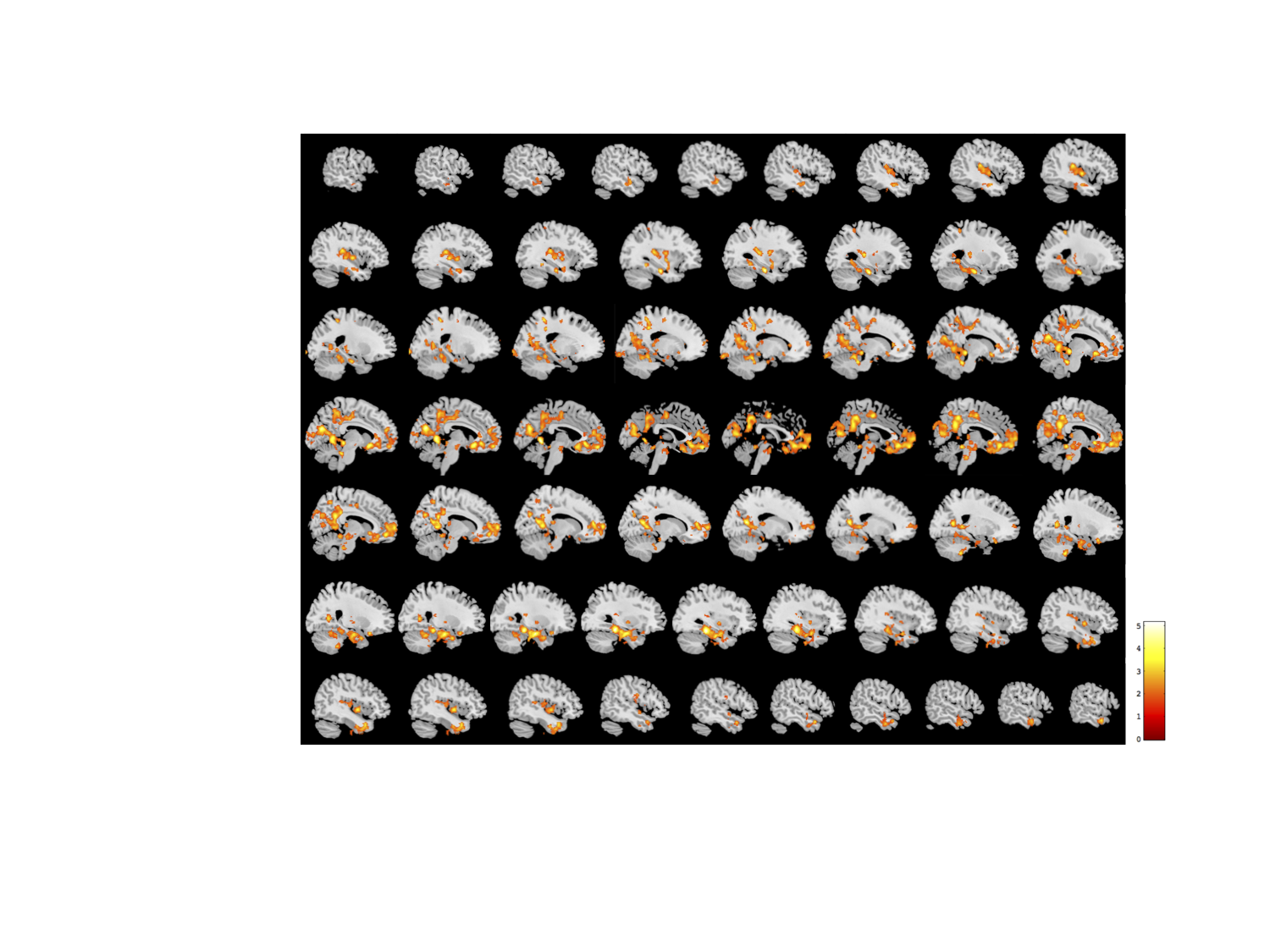

Supplement: Supplementary file 3 [file brb30004-0337-SD3.tiff]

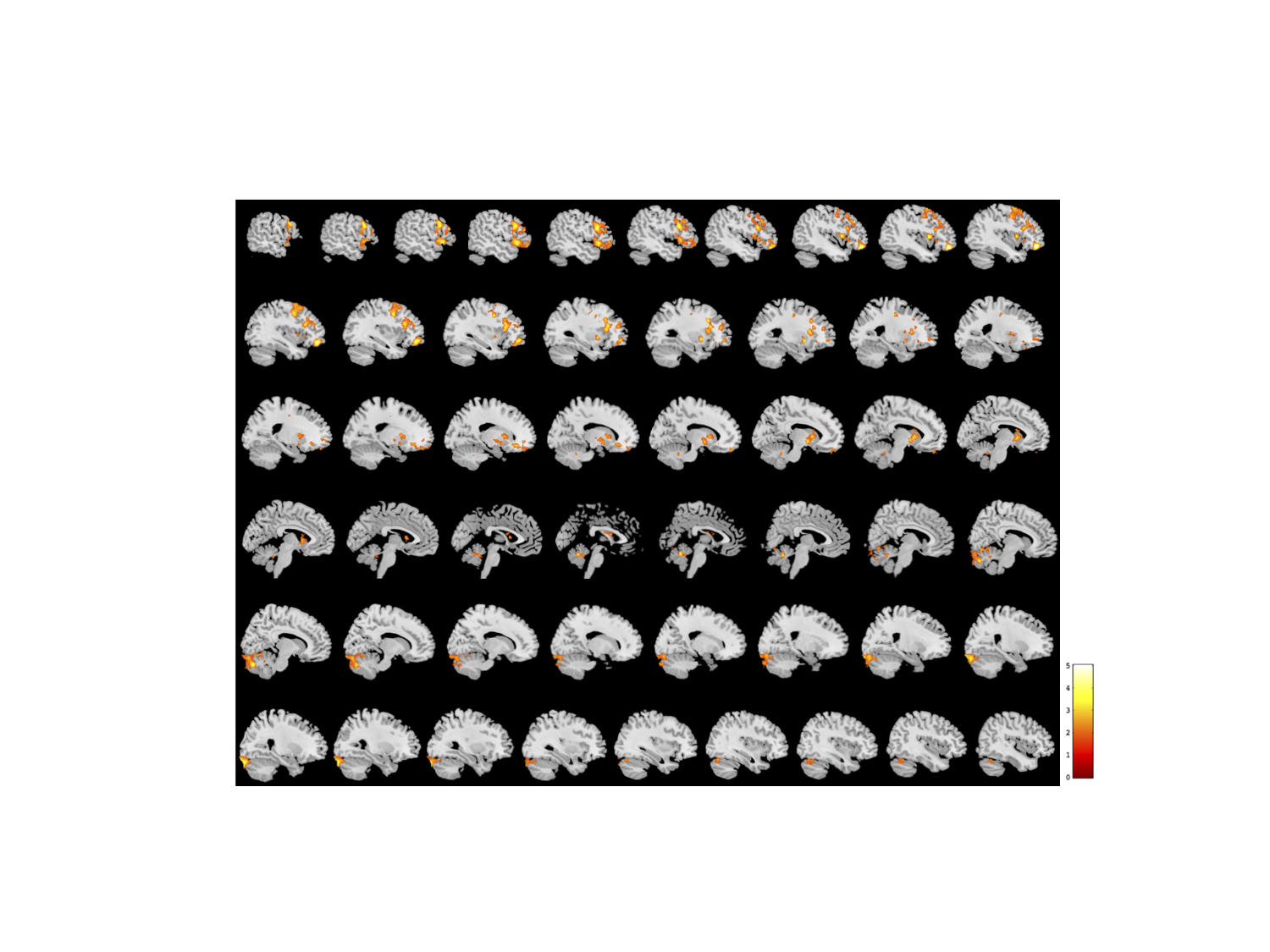

Supplement: Supplementary file 4 [file brb30004-0337-SD4.tiff]
