# Supplementary material for: BOLD signal and functional connectivity associated with loving kindness meditation
Source: Brain Behav. 2014 Feb 12;4(3):337–47. doi: 10.1002/brb3.219 (PMC4055184; doi:10.1002/brb3.219)
Supplement: Supplementary file 5 [file brb30004-0337-SD5.docx]

Table S1. Brain regions showing less intrinsic connectivity during loving kindness meditation in meditators as compared to controls.

Cluster

Brain Region Brodmann Area Side T X Y Z

Cluster 1 (2149 voxels)

Fusiform Gyrus R 5.33 30 -58 -12

Calcarine Gyrus L 5.07 -8 -54 6

Lingual Gyrus 18 L 5.02 -10 -54 -4

Calcarine Gyrus R 4.71 12 -58 16

Parahippocampal Gyrus Hipp (SUB) L 4.68 -18 -40 -6

Precuneus R 4.65 18 -54 18

Cuneus R 4.54 8 -70 28

Cluster 2 (1229 voxels)

Middle Occipital Gyrus L 5.22 -34 -84 20

Middle Temporal Gyrus L 4.1 -42 -64 12

Superior Occipital Gyrus L 4.03 -22 -78 38

Inferior Occipital Gyrus L 3.99 -42 -68 -6

Inferior Parietal Lobule IPC (PGp) L 3.97 -28 -80 46

Cluster 3 (1149 voxels)

Superior Parietal Lobule SPL (7P) R 4.33 10 -82 48

Superior Occipital Gyrus R 4.19 24 -78 24

Middle Occipital Gyrus R 4.19 34 -78 18

Inferior Temporal Gyrus R 4.18 46 -70 -8

Cluster 4 (745 voxels)

Inferior Frontal Gyrus p. Opercularis R 4.41 62 20 20

Inferior Frontal Gyrus p. Triangularis R 4.19 48 44 -2

Middle Orbital Gyrus R 4.17 22 40 -18

Inferior Frontal Gyrus p. Orbitalis R 4.15 26 24 -18

Cluster 5 (614 voxels)

Precuneus SPL (7A) L 4.71 -14 -60 58

Postcentral Gyrus 1 L 4.32 -32 -42 66

Superior Parietal Lobule 1/SPL (7A) L 4.26 -28 -46 70

Inferior Parietal Lobule 2 L 3.42 -50 -38 54

Cluster 6 (600 voxels)

Inferior Frontal Gyrus p. Triangularis L 4.65 -46 30 4

Inferior Frontal Gyrus p. Orbitalis L 4.15 -50 40 -8

Insula Lobe L 4.13 -30 24 12

Cluster 7 (455 voxels)

Postcentral Gyrus 2 R 4.41 56 -20 36

Supramarginal Gyrus IPC (PFt) R 3.73 60 -18 28

Inferior Parietal Lobule 1/2/IPC (PFt) R 3.72 50 -34 56

Cluster 8 (371 voxels)

Supplementary Motor 6 R 5.83 6 8 66

Superior Frontal Gyrus 6 R 3.84 22 -4 72

Supplementary Motor 6 L 3.34 0 12 50

Cluster 9 (367 voxels)

Parahippocampal Gyrus R 4.52 32 -34 -14

Hippocampus Hipp (CA/FD) R 4.35 32 -12 -18

Fusiform Gyrus R 4.19 38 -26 -24

Table S1 continued.

Cluster

Brain Region Brodmann Area Side T X Y Z

Cerebellum R 3.1 28 -24 -30

Cluster 10 (342 voxels)

Middle Cingulate Cortex 4a R 5.04 8 -30 48

Postcentral Gyrus SPL (5L)/3b R 4.07 16 -42 62

Precuneus 3b /SPL (5M) R 3.46 12 -44 66

Paracentral Lobule 4a R 3.29 8 -28 78

Cluster 11 (251 voxels)

Supramarginal Gyrus IPC (PF/PFcm) R 4.72 56 -38 24

Superior Temporal Gyrus IPC (PF) R 4.39 66 -40 14

Middle Temporal Gyrus R 4.39 68 -42 8

Cluster 12 (228 voxels)

Middle Cingulate Cortex L 4.17 -6 2 34

Anterior Cingulate Cortex L 4.14 -6 8 30

Middle Cingulate Cortex R 3.55 2 -4 38

SMA 6 R 3.36 6 -10 52

Cluster 13 (207 voxels)

Superior Orbital Gyrus L 4.14 -18 64 -4

Mid Orbital Gyrus L 3.88 -4 58 -14

Superior Frontal Gyrus L 3.53 -14 66 6

Superior Medial Gyrus L 3.11 -10 66 2

Cluster 14 (185 voxels)

Inferior Frontal Gyrus p. Orbitalis R 4.97 52 22 -8

Insula Lobe R 3.9 36 30 0

Inferior Frontal Gyrus p. Opercularis R 3.56 56 20 -2

Inferior Frontal Gyrus p. Triangularis R 3.2 38 28 8

*p*<.05 FWE, cluster-corrected

Table S2. Brain regions showing greater functional connectivity with the posterior cingulate cortex/precuneus during loving kindness meditation in novices as compared to meditators.

Cluster

Brain Region Brodmann Area Side T X Y Z

Cluster 1 (12153 voxels)

Parahippocampal Gyrus Hipp (CA) R 5.14 30 -32 -14

Hippocampus Hipp (CA/FD) L 5.02 -32 -16 -22

Cerebellum Lobule V L 4.97 -4 -52 -2

Hippocampus Hipp (CA/FD)/Amyg R 4.96 28 -10 -22

Precuneus 18 R 4.93 16 -58 14

Calcarine Gyrus L 4.76 -6 -72 22

Lingual Gyrus L 4.19 -10 -32 -6

Posterior Cingulate Cortex R 4.17 6 -48 28

Cerebellum Lobules I-IV (Hem) L 4.14 -12 -38 -26

Parahippocampal Gyrus Hipp (CA/SUB/FD) R 4 32 -14 -26

Precuneus R 3.89 4 -44 42

Cluster 2 (3607 voxels)

Olfactory cortex L 4.36 -6 20 -12

Mid Orbital Gyrus R 4.12 2 38 -10

Olfactory cortex R 4.07 6 18 -6

Mid Orbital Gyrus L 3.97 -4 56 -14

Anterior Cingulate Cortex L 3.8 -4 32 -6

Superior Medial Gyrus R 3.75 10 66 6

Mid Orbital Gyrus R 3.56 8 52 -8

Anterior Cingulate Cortex R 3.53 14 48 12

Inferior Frontal Gyrus (p. Orbitalis) R 3.44 22 22 -20

Superior Medial Gyrus R 3.33 10 52 6

Anterior Cingulate Cortex L 3.27 0 48 12

*p*<.05 FWE, cluster-corrected

Table S3. Brain regions showing greater functional connectivity with the posterior cingulate cortex/precuneus during loving kindness meditation in meditators as compared to novices.

Cluster

Brain Region Brodmann Area Side T X Y Z

Cluster 1 (3783 voxels)

Inferior Frontal Gyrus p. Orbitalis L 5.02 -42 46 -12

Inferior Frontal Gyrus p. Opercularis L 3.91 -42 8 8

Inferior Frontal Gyrus p. Orbitalis L 3.79 -52 20 -4

Inferior Frontal Gyrus p. Triangularis L 3.78 -48 20 24

Inferior Frontal Gyrus p. Opercularis L 3.7 -56 14 32

Middle Frontal Gyrus L 3.68 -30 22 32

Precentral Gyrus L 3.55 -36 2 44

Inferior Frontal Gyrus p. Triangularis L 3.52 -36 24 24

Insula Lobe L 3.5 -28 18 14

Putamen L 3.43 -30 8 -4

Cluster 2 (1369 voxels)

Cerebellar Vermis Lobule V/VI 3.99 2 -58 -22

Lingual Gyrus 17/18 R 3.99 26 -98 -18

Cerebellum Lobule VIIa Crus I R 3.87 10 -76 -32

Cerebellum R 2.92 10 -86 -20

Lingual Gyrus Lobule VI (Hem) R 2.74 8 -74 -12

Cerebellum Lobule VIIa Crus I R 2.66 20 -90 -32

Cerebellum Lobule VIIa Crus I R 2.61 12 -84 -24

Cerebellum Lobule VIIa Crus I R 2.58 22 -78 -22

*p*<.05 FWE, cluster-corrected

**Figure S1.** All slices are displayed for the comparison of BOLD signal during loving kindness meditation between meditators and novices, corresponding to Figure 1. Brain regions in blue/cold show reduced BOLD signal during loving kindness meditation in meditators as compared to novices (*p*<.05 FWE, cluster-corrected; slices displayed left to right).

**Figure S2.** All slices are displayed for the comparison of intrinsic connectivity during loving kindness meditation between meditators and novices, corresponding to Figure 2. Brain regions in blue/cold show less intrinsic connectivity during loving kindness meditation in meditators as compared to novices (*p*<.05 FWE, cluster-corrected; slices displayed left to right).

**Figure S3.** All slices are displayed for the comparison of seed-based functional connectivity during loving kindness meditation for novices greater than meditators. Brain regions in yellow/hot show greater functional connectivity with the posterior cingulate cortex/precuneus during loving kindness meditation in novices than meditators (*p*<.05 FWE, cluster-corrected; slices displayed left to right).

**Figure S4.** All slices are displayed for the comparison of seed-based functional connectivity during loving kindness meditation for meditators greater than novices. Brain regions in yellow/hot show greater functional connectivity with the posterior cingulate cortex/precuneus during loving kindness meditation in meditators than novices (*p*<.05 FWE, cluster-corrected; slices displayed left to right).
